# Endophytic Fungi Inoculation Reduces Ramulosis Severity in *Gossypium hirsutum* Plants

**DOI:** 10.3390/microorganisms12061124

**Published:** 2024-05-31

**Authors:** Isabella de Oliveira Silva, Layara Alexandre Bessa, Mateus Neri Oliveira Reis, Damiana Souza Santos Augusto, Charlys Roweder, Edson Luiz Souchie, Luciana Cristina Vitorino

**Affiliations:** 1 Laboratory of Agricultural Microbiology, Federal Institute Goiano, Rio Verde Campus, Rio Verde 75901-970, Brazil; isabella.oi@outlook.com (I.d.O.S.); edson.souchie@ifgoiano.edu.br (E.L.S.); 2 Simple Agro Corporation, 400 Parque General Borges Forte St., Jardim Goiás, Rio Verde 75903-421, Brazil; layara.bessa@ifgoiano.edu.br (L.A.B.); damianarv2012@hotmail.com (D.S.S.A.); 3 Laboratory of Metabolism and Genetics of Biodiversity, Federal Institute Goiano, Rio Verde Campus, Rio Verde 75901-970, Brazil; mateusnerioliveira@hotmail.com; 4 Laboratory of Silviculture and Forestry Production, Federal Institute Goiano, Rio Verde Campus, Rio Verde 75901-970, Brazil; charlys.roweder@ifgoiano.edu.br

**Keywords:** biotic stress, biocontrol, phytopathogenic fungi, fungal diseases, antibiosis

## Abstract

Biotic stress in cotton plants caused by the phytopathogenic fungus *Colletotrichum gossypii* var. *cephalosporioides* triggers symptoms of ramulosis, a disease characterized by necrotic spots on young leaves, followed by death of the affected branch’s apical meristem, plant growth paralysis, and stimulation of lateral bud production. Severe cases of ramulosis can cause up to 85% yield losses in cotton plantations. Currently, this disease is controlled exclusively by using fungicides. However, few studies have focused on biological alternatives for mitigating the effects of contamination by *C. gossypii* var. *cephalosporioides* on cotton plants. Thus, the hypothesis raised is that endophytic fungi isolated from an Arecaceae species (*Butia purpurascens*), endemic to the Cerrado biome, have the potential to reduce physiological damage caused by ramulosis, decreasing its severity in these plants. This hypothesis was tested using plants grown from seeds contaminated with the pathogen and inoculated with strains of *Gibberella moniliformis* (BP10EF), *Hamigera insecticola* (BP33EF), *Codinaeopsis* sp. (BP328EF), *G. moniliformis* (BP335EF), and *Aspergillus* sp. (BP340EF). *C. gossypii* var. *cephalosporioides* is a leaf pathogen; thus, the evaluations were focused on leaf parameters: gas exchange, chlorophyll a fluorescence, and oxidative metabolism. The hypothesis that inoculation with endophytic strains can mitigate physiological and photochemical damage caused by ramulosis in cotton was confirmed, as the fungi improved plant growth and stomatal index and density, increased net photosynthetic rate (*A*) and carboxylation efficiency (*A*/*Ci*), and decreased photochemical stress (ABS/RC and DI_0_/RC) and oxidative stress by reducing enzyme activity (CAT, SOD, and APX) and the synthesis of malondialdehyde (MDA). Control plants developed leaves with a low adaxial stomatal index and density to reduce colonization of leaf tissues by *C. gossypii* var. *cephalosporioides* due to the absence of fungal antagonism. The *Codinaeopsis* sp. strain BP328EF can efficiently inhibit *C. gossypii* var. *cephalosporioides* in vitro (81.11% relative inhibition), improve gas exchange parameters, reduce photochemical stress of chlorophyll-*a*, and decrease lipid peroxidation in attacked leaves. Thus, BP328EF should be further evaluated for its potential effect as a biological alternative for enhancing the resistance of *G. hirsutum* plants and minimizing yield losses caused by *C. gossypii* var. *cephalosporioides*.

## 1. Introduction

Plants under biotic stress experience metabolic disturbances induced by pathogenic microorganisms [1]. These disturbances can lead to diseases with a variety of symptoms, including chlorosis, wilting, localized lesions, and large necroses. Currently, cotton (*Gossypium hirsutum* L.) is one of the most affected crops by biotic stress, as many pests, such as insects and phytopathogenic microorganisms, attack plants throughout their cycle, causing damage to different plant parts and resulting in significant economic losses [2]. Cotton is the world’s leading source of natural fibers, with an estimated planted area of more than 32 million hectares in the 2023–2024 crop season [3], although the negative impacts of pathogen attacks gradually increase global production costs [4]. Bacteria and fungi that attack leaves, stems, roots, and fruits are among the most important causes of cotton diseases. 

The pathogenic fungi include *Fusarium oxysporum* f. sp. *vasinfectum*, which causes Fusarium wilt [5]; *Verticillium dahliae*, which causes Verticillium wilt [6]; *Mycosphaerella areola*, which causes Ramularia leaf spot [7]; *Sclerotium rolfsii* and *Rhizoctonia solani*, which cause root rot [8]; and *Colletotrichum gossypii* and *Colletotrichum gossypii* var. *cephalosporioides*, which cause anthracnose and ramulosis, respectively [9,10].

The main cotton-producing regions in South America are affected by *C. gossypii* var. *cephalosporioides*. Severe outbreaks cause significant reductions in production, often associated with meristem necrosis, excessive sprouting, branching, and stunting [11]. This fungus infects leaves, petioles, and stems, hindering bract formation and, consequently, cotton yield [12]. Damage caused by ramulosis to cotton crops varies from 20% to 30%, reaching 85% in severe cases [13]. This disease has been exclusively controlled using chemical products, which leads to disease outbreaks by promoting pathogen resistance. Persistent challenges associated with the use of traditional fungicides also include toxicity to humans and non-target organisms, as well as environmental pollution [14]. Thus, the development of biological alternatives for ramulosis management in cotton plantations has been encouraged. In this context, the use of endophytic fungi is a promising option, as several species act as biocontrol agents against pests and diseases, enabling a sustainable suppression of phytopathogens [15,16,17]. Many endophytic fungi are potential resources for biocontrol, as they reduce the effects or prevent diseases in plants not only through direct or indirect antibiosis but also by promoting growth and improving resistance in host plants [18]. On the other hand, tests need to be seriously conducted, as endophytic isolates can, under some conditions, exert negative effects on the host plant [19].

Endophytic fungi have been tested for biocontrol of important cotton diseases such as Verticillium wilt e.g., [20,21,22] and root rot e.g., [23]. However, few studies have been conducted under in vitro conditions, focusing on the biological control of *C. gossypii* [24,25], and even fewer under greenhouse or field conditions. Investigations into alternative biological perspectives for controlling *C. gossypii* var. *cephalosporioides* are also scarce e.g., [26]. 

Studies have shown that *C. gossypii* var. *cephalosporioides* is transmitted externally and internally by seeds, which are the most efficient dissemination agents. Seeds carry this pathogen over short and long distances, leading to the introduction of ramulosis in new areas [27]. Therefore, cotton seeds were infected by *C. gossypii* var. *cephalosporioides* to test the hypothesis that endophytic fungi isolated from an Arecaceae species (*Butia purpurascens*), which is endemic to the Cerrado biome [28], have the potential to mitigate physiological damage caused by ramulosis, decreasing its severity in *G. hirsutum* plants. The present study contributes to the search for conservation strategies based on the use of available biodiversity resources. In this context, the Cerrado biome has been an important source of microorganisms of biotechnological interest that can establish symbiotic relationships with agricultural species [29,30]. The use of strains from the Cerrado microbiota for producing biocontrol agents promotes the valorization of the biome’s biodiversity and awareness for its conservation.

*C. gossypii* var. *cephalosporioides* is a hemibiotrophic pathogen that initially infects through a biotrophic stage, associated with large primary intracellular hyphae, and subsequently in the necrotrophic stage, when the fungus causes significant changes in the cotton physiology due to secretion of lytic enzymes and nonspecific toxins [12], as narrower secondary hyphae spread throughout the host’s tissues [31]. From a physiological perspective, photosynthetic processes such as gas exchange and chlorophyll fluorescence are among the most damaged by pathogens that infect leaves, such as *C. gossypii* var. *cephalosporioides* [32]. Several studies have shown that pathogen infections lead to reduced photosynthesis [33,34,35] and changes in photosystems [36]. These plants are affected by mesophyll cell damage, colonization of intra- and intercellular spaces, and stomatal closure, affecting transpiration, CO_2_ influx, and photosynthetic rate [37,38]. Thus, cotton plants biotically stressed by *C. gossypii* var. *cephalosporioides* and, consequently, exhibiting ramulosis symptoms were chosen for evaluation to better understand the effects of inoculation with endophytic fungi on the physiology of these plants.

Besides the biocontrol of cotton diseases, endophytic fungi can increase the availability of organic cotton in the market, whose production is encouraged by consumer interests and industry certification standards [39]. Organic fibers are used in several products, and organic cottonseeds are utilized in animal feed and organic oil manufacturing. Therefore, induced resistance and biological control resulting from inoculation are expected to minimize impacts caused by biotic stress in cotton plants, as it is a key practice in sustainable agriculture, not only to control diseases caused by phytopathogens but also to reduce production costs [40]. Thus, the objective of this study was to assess the effect of endophytic fungi inoculation on plant growth, gas exchange, photochemistry, and oxidative stress of *G. hirsutum* plants infected by *C. gossypii* var. *cephalosporioides* and, therefore, ramulosis exhibiting symptoms. This study focused on developing an alternative for minimizing losses caused by *C. gossypii* in cotton yield by improving plant performance under biotic stress.

## 2. Materials and Methods

### 2.1. Isolated Fungi and Seeds Contaminated with C. gossypii var. cephalosporioides

Tests were conducted using root-endophytic strains isolated from *Butia purpurascens* (Arecaceae). These strains are currently part of the culture collection at the Agricultural Microbiology Laboratory of the Federal Institute Goiano, Rio Verde, GO, Brazil. The strains were cultured on Potato Dextrose Agar medium (infusion of 200 g of potato, 20 g of dextrose, and 15 g of agar) for 7 days at 30 °C to obtain replicates of each culture. The evaluated strains were: BP10EF (*Gibberella moniliformis*), BP33EF (*Hamigera insecticola*), BP328EF (*Codinaeopsis* sp.), BP335EF (*Gibberella moniliformis*), and BP340EF (*Aspergillus* sp.). These strains were chosen because they exhibited antibiosis to *C. gossypii* var. *cephalosporioides* in previously conducted tests (see Section 2.2).

**Figure 1:**
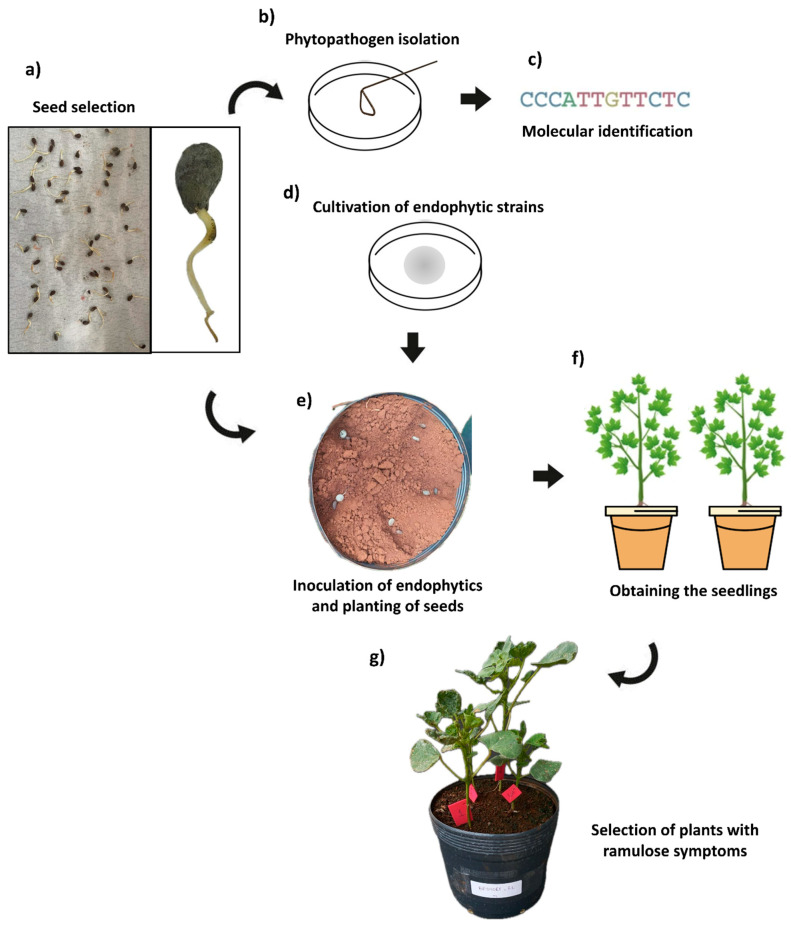
Procedures used to obtain *Gossypium hirsutum* plants with ramulosis symptoms and inoculated with endophytic fungal strains. Obtaining seeds colonized by *Colletotrichum gossypii* var. *cephalosporioides* (**a**); isolation and identification of the phytopathogen (**b**,**c**); cultivation of endophytic fungal strains (**d**); exposure of *Gossypium hirsutum* seeds to endophytic fungi (**e**); obtaining seedlings (**f**); and selection of symptomatic seedlings for ramulosis (**g**).

Cotton seeds contaminated with *C. gossypii* var. *cephalosporioides* were obtained through phytosanitary quality tests. Seeds of the variety TMG47-B2RF/2021 from 4 seed lots, free from treatment with fungicide or insecticide, were evaluated on germination paper. They were arranged on paper sheets moistened with distilled water, covered with plastic film, and placed in a BOD chamber, where they remained for 4 days at 35 °C (Figure 1a). Infestation with *C. gossypii* var. *cephalosporioides* was confirmed in 63% of the seeds, and one of the seed lots was used for the in vivo experiment.

Three seeds contaminated with *C. gossypii* var. *cephalosporioides* were aseptically placed on a plate containing PDA medium. These seeds were used to establish fungal cultures, which were purified and taken to the Biological Institute of São Paulo for molecular identification (Figure 1b,c). All cultures were molecularly identified as belonging to the species *C. gossypii* var. *cephalosporioides*. The identification was performed by partial sequencing of the internal transcribed spacer and the calmodulin and β-tubulin genes. For this, amplicons of 575, 532, and 478 nucleotides were obtained for these respective genes. Sequencing was performed using the Sanger method; for phylogenetic inference, sequences were paired by similarity to sequences in GenBank using BLASTn while considering homology greater than 99%.

### 2.2. In Vitro Antibiosis Tests

In vitro antibiosis tests using the paired culture technique were performed to better understand the interaction between *C. gossypii* var. *cephalosporioides* and the endophytic strains tested. Thus, 5-cm-diameter mycelial discs of the phytopathogen and endophytic fungi were placed equidistantly on plates containing BDA medium. These plates were then incubated at 30 °C and left to rest for 72 h, when the diameters of colony halos were measured. Plates with the phytopathogen, without inoculation with the endophytic strains, were used as controls.

The test was conducted in triplicate for each endophytic strain tested, and the data were used to estimate the percentage of inhibition of phytopathogenic fungus growth induced by the endophytic strains. This was calculated through the relative inhibition index (RII):

$$\mathrm{RII}\;(100\%)=\frac{\mathrm{RC}-\mathrm{RX}}{\mathrm{RC}}\times 100$$

where

RC = radius of the phytopathogen colony in the control treatment; and

RX = radius of the phytopathogen colony paired with the endophytic strain.

### 2.3. Preparation of the Plant Substrate and Seed Planting 

The experiment was conducted in a greenhouse at the Tissue Culture Laboratory of the Federal Institute Goiano, in Rio Verde, GO, Brazil, from June to August 2022, at mean air temperature of 30.26 °C and relative air humidity of 29.43%.

The seeds were sanitized before planting through the asepsis process described by Reis et al. [41] to remove epiphytic microorganisms and ensure that only the endogenous contaminant *C. gossypii* var. *cephalosporioides* remained. The seeds were left to rest for 30 min and then planted in 3-kg pots containing a mixture of soil and nutritional substrate (Bioplant Garden^®^, Bio Plant Life, Santa Ana, CA, USA) at concentrations of 70% and 30%, respectively. This mixture was previously sterilized at 121 °C for 30 min to avoid interaction of seeds with microorganisms and subsequently kept in impermeable bags; it was placed in the pots only at the time of planting.

Five seeds were sown per pot, arranged in 3-cm-deep furrows; 5-mm-diameter mycelial discs of the tested endophytic fungi were inoculated directly onto the seeds to provide a simultaneous development of hyphae and radicles (Figure 1d,e).

The plants were evaluated daily for visual symptoms of ramulosis up to the V2 developmental stage. Some plants presented disease symptoms at the V0 stage (cotyledon) 14 days after planting, presenting small, dark, circular necrotic lesions, like those described by Talhinhas and Baroncelli [31] for lesions caused by *C. gossypii* var. *cephalosporioides* in the early stages of cotton development. Most plants presented differentiated leaf development and wrinkling of leaf blades 21 days after planting. Thinning was carried out at this time, maintaining only seedlings with ramulosis symptoms in the pots (two plants per pot) (Figure 1f,g). Emergence of leaf lesions, followed by a halt in branch growth, as well as emergence of new lateral buds, were found 30 days after planting. These new branches tended to form clusters characterized by an excess of nodes and internodes, resulting in plants with a bushy appearance, consistent with those described by Araújo [27] for infections by *C. gossypii* var. *cephalosporioides* in cotton plants. Thus, the occurrence of ramulosis was confirmed in all evaluated plants. The plants were irrigated daily according to their needs over the experimental period.

### 2.4. Biometric, Gas Exchange, and Chlorophyll-a Fluorescence Evaluations 

Biometric and physiological evaluations were performed 30 days after planting, when ramulosis symptoms were confirmed in all cotton plants, at the vegetative phenological stage. Data on plant height (cm), stem diameter (cm), number of leaves, and shoot fresh and dry weights (g) were obtained. Dry weight was obtained after drying the plants in a forced air circulation oven at 65 °C until constant weight.

Physiology and antioxidant metabolism analyses were performed when the plants reached the V5 stage. Gas exchanges were evaluated using an infrared gas analyzer equipped with a fluorometer (LI-6400xt; LI-COR, Lincoln, NE, USA) to determine net photosynthetic rate (*A*; μmol m^−2^ s^−1^); intercellular CO_2_ concentration (*Ci*); stomatal conductance of water vapor (*gs*); transpiration rate (*E* mmol m^−2^ s^−1^); and the ratio of intercellular to ambient CO_2_ concentration (*Ci/Ca*). Measurements were always made on the youngest fully expanded leaf facing the sun, between 08:00 h and 11:00 h, using constant photosynthetically active radiation (1000 μmol photons m^−2^ s^−1^), with records of atmospheric CO_2_ concentration, relative air humidity, air temperature, and radiation. The carboxylation efficiency of plants was calculated using *A*/*Ci*.

The OJIP chlorophyll-*a* fluorescence was estimated using a portable fluorometer (FluorPen FP 100; Photon Systems Instruments, Drasov, Czech Republic). The fourth leaf of all sampled units was previously dark-adapted for 30 min for complete oxidation of the photosynthetic electron transport system and then subjected to a 3000 µmol m^−2^ s^−1^ pulse of blue light to measure the minimum fluorescence (F_0_) at 50 μs, when all photosystem II (PSII) reaction centers are open and defined as step O, followed by step J (2 ms), step I (30 ms), and maximum fluorescence (F_M_), when all PSII reaction centers are closed, known as step P. These values were used to estimate several bioenergetic indices of PSII, according to Strasser et al. [42]. 

The parameters estimated were: relatively low values of specific light absorption flux per active reaction center (ABS/RC); trapped per reaction center (TR_0_/RC); electron transport flux per reaction center (ET_0_/RC); specific energy dissipation flux at the antenna chlorophyll level (DI_0_/RC); photosynthetic performance index (PI_ABS_), which incorporates the cascade of energy events from initial absorption to PQ reduction; maximum quantum yield of primary photochemistry (PHI_P0_); quantum yield of energy dissipation (PHI_D0_); and quantum yield of electron transport (PHI_E0_).

### 2.5. Extraction and Activity of Antioxidant Metabolism Enzymes and Malondialdehyde (MDA)

The activity of enzymes from the antioxidant and lipid peroxidation systems was quantified. Samples were collected, placed in liquid nitrogen, and stored in an ultrafreezer at −80 °C. 

Enzyme extraction was carried out as follows: 200 mg of leaf tissues were macerated in liquid nitrogen with 50% PVPP and following the extraction protocol proposed by Biemelt et al. [43], with an extraction buffer composed of 100 mM potassium phosphate (pH 7.8), 0.1 mM EDTA, and 10 mM ascorbic acid. The extract was centrifuged at 13,000× *g* for 10 min at 4 °C, and the supernatant was used to evaluate the activity of catalase (CAT), ascorbate peroxidase (APX), guaiacol peroxidase (POD), and superoxide dismutase (SOD).

CAT activity was evaluated according to the methodology proposed by Havir and McHale [44]: An aliquot of the enzyme extract was added to an incubation medium containing 100 mM potassium phosphate (pH 7.0) and 12.5 mM hydrogen peroxide. Enzyme activity was determined based on the consumption of H_2_O_2_ every 15 s for 3 min at 240 nm in a spectrophotometer. The molar extinction coefficient used was 36 mM^−1^ cm^−1^. CAT activity was expressed as µmol H_2_O_2_ min^−1^ mg^−1^ protein. 

APX activity was evaluated using the methodology of Nakano and Asada [45], considering an ascorbate oxidation rate of 290 nm every 15 s for 3 min. An aliquot of the enzyme extract was added to a medium containing 100 mM potassium phosphate buffer (pH 7.0), 0.5 mM ascorbic acid, and 0.1 mM peroxide of hydrogen. The molar extinction coefficient used was 2.8 mM^−1^ cm^−1^. APX activity was expressed as µmol AsA min^−1^ mg^−1^ protein. 

POD activity was evaluated using the methodology of Fang and Kao [46], considering the formation of tetraguaiacol by the increase in absorbance. An aliquot of the enzyme extract was added to a medium containing 50 mM sodium phosphate buffer (pH 6.0) and 0.13% guaiacol; 0.15% H_2_O_2_ was added before the spectrophotometric readings at 470 nm for 3 min. The molar extinction coefficient used was 26.6 mM^−1^ cm^−1^. POD activity was expressed as µmol H_2_O_2_ min^−1^ mg^−1^ protein. Proteins from leaf samples were quantified according to the Bradford method [47]. The absorbances were read at 595 nm, and the final data were used to express the enzyme activities.

SOD activity was determined based on the methodology of Giannopolitis and Ries [48], considering the enzyme’s ability to inhibit photoreduction of nitro-blue tetrazolium (NBT). An aliquot of the extract was incubated in a medium containing 50 mM potassium phosphate (pH 7.8), 14 mM methionine, 0.1 µM EDTA, 75 µM NBT, and 2 µM riboflavin. The samples, along with the incubation medium, were illuminated with a 20-W fluorescent lamp for 7 min. Readings were taken with a spectrophotometer at 560 nm. SOD activity was expressed as U mg^−1^ protein (1U = quantity of enzymes needed to inhibit NBT photoreduction by 50%).

Malondialdehyde (MDA) was quantified by macerating 200 mg of leaf tissue in liquid nitrogen and PVPP, followed by homogenization in 0.1% (m v^−1^) trichloroacetic acid (TCA) and centrifugation at 10,000× *g* for 15 min at 4 °C. The amount of MDA was determined using the methodology proposed by Buege and Aust [49].

### 2.6. Experimental Design and Statistical Analyses

The in vitro antibiosis experiment was conducted in a completely randomized experimental design with 5 treatments (exposure of the phytopathogen to five endophytic fungal strains), and the greenhouse experiment was conducted in a randomized block design with 6 treatments (5 endophytic fungal strains and a control). Plants inoculated with culture medium discs without mycelium were used as controls. The experiments were conducted with five replications per treatment, considering two plants per pot as one replication, totaling 60 units. The data obtained for the treatments were subjected to a one-way ANOVA to evaluate the effect of inoculation with endophytic strains. When the effects were significant, the means were evaluated by Tukey’s test at a 5% significance level. Subsequently, all variables that showed significant differences were jointly evaluated in a correlation matrix and connected by means of principal component analysis (PCA). Considering that these variables had different units of measurement, PCA was recovered using standardized data to obtain a mean of 0 and a standard deviation of 1. The number of principal components was defined according to eigenvalues (>1.0) and variance explained (>70%). The statistical tests were conducted using the R 4.3.2 program [50].

The similarity matrix was developed to estimate similarities or differences among plants from different treatments. The similarity index was obtained using the Pearson correlation coefficient, with values of *r* transformed into *d* = (1 − *r*) × 100 to estimate the distance (*d*). A dendrogram was then recovered using the unweighted pair group method with arithmetic mean (UPGMA), with an adjustment between the distance matrix and the dendrogram estimated by the cophenetic correlation coefficient [51]. This analysis was conducted using the DendroUPGMA program [52].

## 3. Results

### 3.1. Antibiosis of Endophytic Strains of Colletotrichum gossypii var. cephalosporioides

**Figure 2:**
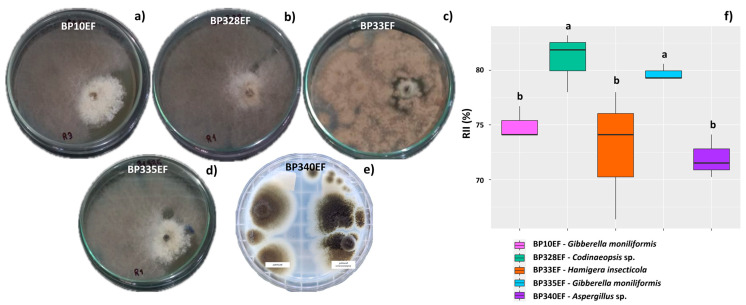
Compatibility between *Colletotrichum gossypii* var. *cephalosporioides* and the endophytic fungi: BP10EF: *Gibberella moniliformis* (**a**), BP33EF: *Hamigera insecticola* (**b**), BP328EF: *Codinaeopsis* sp. (**c**), BP335EF: *Gibberella moniliformis* (**d**), and BP340EF: *Aspergillus* sp. (**e**). Antibiosis of endophytic fungi to *C. gossypii* var. *cephalosporioides* in the paired colony test (**a**) and relative inhibition index (%) (**f**). In (**a**–**e**), the colonies on the left are the endophytic fungi, and those on the right are the phytopathogens. In (**f**), black horizontal bars within the boxplots represent the median. Vertical bars show the maximum and minimum values, and the points outside the box are outlier values. Equal letters above the boxes represent statistically equal means (Tukey’s test, *p* < 0.05).

The antibiosis test showed expressive mycelial development of all endophytic fungal strains in relation to the phytopathogen, which resulted in the inhibition of growth of *C. gossypii* var. *cephalosporioides* (Figure 2a–e). The estimated relative inhibition index (RII) confirmed the potential of all endophytic strains to inhibit the in vitro growth of *C. gossypii* var. *cephalosporioides*, as the mean RIIs found were, in general, higher than 72% (Figure 2b). However, the comparison of strains showed that BP328EF (*Codinaeopsis* sp.) and BP335 (*Gibberella moniliformis*) were more effective in inhibiting the phytopathogen, showing the highest RII means (81.11% and 79.81%, respectively).

### 3.2. Growth and Physiology of Cotton Plants Infected by Colletotrichum gossypii var. cephalosporioides and Subjected to Inoculation with Endophytic Strains 

**Figure 3:**
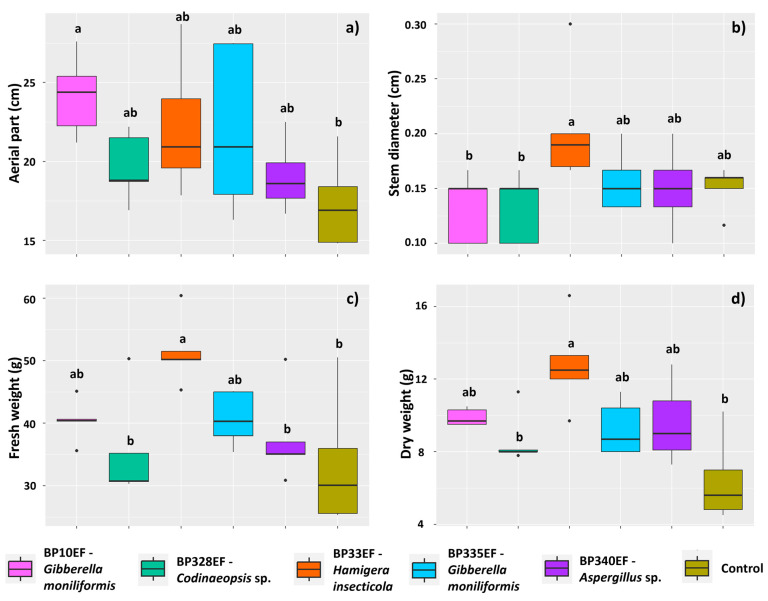
Growth of *Gossypium hirsutum* plants under attack of *Colletotrichum gossypii* var. *cephalosporioides* and inoculated with endophytic fungi. Plant height (cm) (**a**); stem diameter (cm) (**b**); shoot fresh weight (g) (**c**); and shoot dry weight (g) (**d**). Black horizontal bars within the boxplots represent the median. Vertical bars show the maximum and minimum values, and the points outside the box are outlier values. Equal letters above the boxes represent statistically equal means (Tukey’s test, *p* < 0.05).

In general, the inoculation with endophytic fungi positively affected the development of cotton plants infected with *C. gossypii* var. *cephalosporioides*. Control plants presented a lower mean height (17.31 cm) than plants inoculated with BP10EF (24.17 cm) (Figure 3a). Stem diameter was also affected by fungal treatments; the highest means were found for plants inoculated with BP33EF (0.20 cm) (Figure 3b). Shoot fresh weight (33.50 g) and shoot dry weight (6.42 g) of control plants were also lower than those of inoculated plants; the highest means were found for plants inoculated with BP33EF (51.50 and 12.82 g, respectively) (Figure 3c,d).

**Figure 4:**
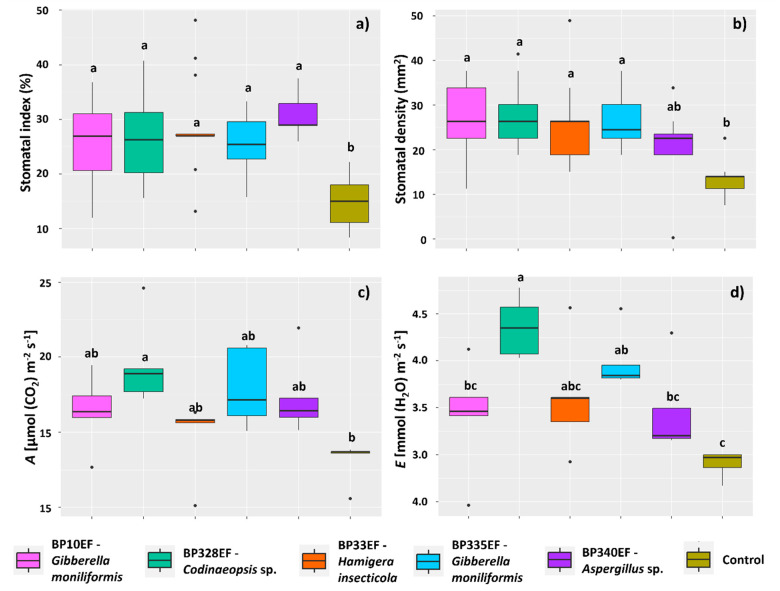
Stomatal parameters on the adaxial leaf surface and gas exchanges in *Gossypium hirsutum* plants under attack of *Colletotrichum gossypii* var. *cephalosporioides* and inoculated with endophytic fungi. Stomatal index (%) (**a**); stomatal density (mm^2^) (**b**); net photosynthetic rate: *A* (**c**); and transpiration rate: *E* (**d**). Black horizontal bars within the boxplots represent the median. Vertical bars show the maximum and minimum values, and the points outside the box are outlier values. Equal letters above the boxes represent statistically equal means (Tukey’s test, *p* < 0.05).

Inoculation with endophytic fungi had no effect on the stomatal index or stomatal density on the adaxial surface of cotton leaves affected by ramulosis; however, on the adaxial surface, control plants had the lowest percentage of stomata (14.96%). However, plants subjected to the different endophytic strains did not show any difference in adaxial stomatal index (Figure 4a). Stomatal density showed similar results, with lower means in control plants (12.87) and in plants inoculated with BP340EF (20.72) (Figure 4b).

Regarding gas exchanges, fungal inoculation, in general, tended to improve the net photosynthetic rate (*A*) in plants affected by ramulosis; however, higher *A* means were found for plants inoculated with BP328EF (19.53 µmol (CO_2_) m^−2^ s^−1^) (Figure 4c). Inoculation with BP328EF, however, significantly increased the transpiration rate (4.36 mmol (H_2_O) m^−2^ s^−1^); control plants had the lowest water loss (2.90 mmol (H_2_O) m^−2^ s^−1^) (Figure 4d).

**Figure 5:**
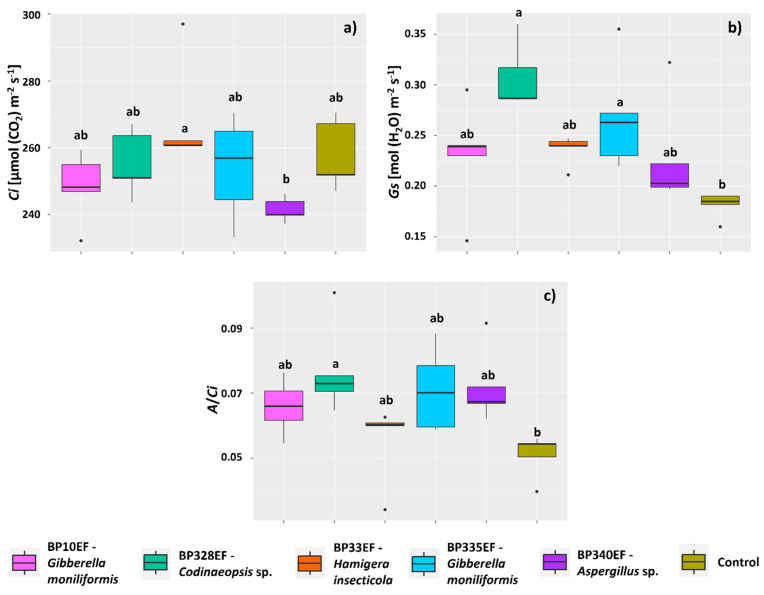
Gas exchange parameters in *Gossypium hirsutum* plants under attack of *Colletotrichum gossypii* var. *cephalosporioides* and inoculated with endophytic fungi. Intercellular CO_2_ concentration: *Ci* (**a**); stomatal conductance: *Gs* (**b**); and carboxylation efficiency: *A*/*Ci* (**c**). Black horizontal bars within the boxplots represent the median. Vertical bars show the maximum and minimum values, and the points outside the box are outlier values. Equal letters above the boxes represent statistically equal means (Tukey’s test, *p* < 0.05).

The inoculation treatments had no effect on intercellular CO_2_ concentration (*Ci*), which showed similar means to the control; however, *Ci* tended to increase in plants inoculated with BP33EF (268.24 µmol (CO_2_) m^−2^ s^−1^) and decrease in plants inoculated with BP340EF (241.49 µmol (CO_2_) m^−2^ s^−1^) (Figure 5a). Stomatal conductance (*Gs*) was higher in plants inoculated with BP328EF and BP335EF (respectively 0.30 and 0.26 mol (H_2_O) m^−2^ s^−1^) compared with control plants (0.23 mol (H_2_O) m^−2^ s^−1^) (Figure 5b). Carboxylation efficiency (*A*/*Ci*) was also affected by inoculation with endophytic strains, tending to be higher in plants inoculated with BP328EF (0.07) and lower in control plants (0.05).

**Figure 6:**
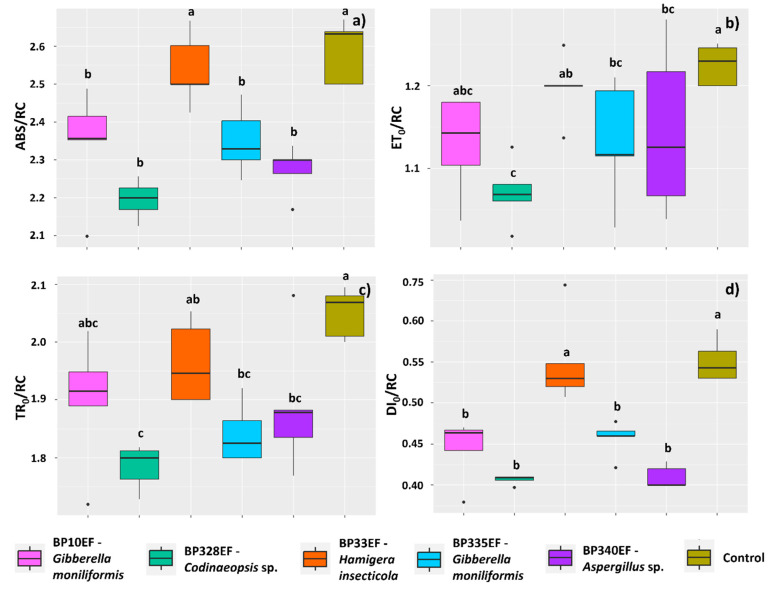
Primary photochemistry by chlorophyll-*a* fluorescence in *Gossypium hirsutum* plants under attack of *Colletotrichum gossypii* var. *cephalosporioides* and inoculated with endophytic fungi. Light absorption flux per active reaction center (ABS/RC) (**a**); electron transport flux per reaction center (ET_0_/RC) at t = 0 (**b**); trapped energy flux per reaction center (TR_0_/RC) at t = 0 (**c**); specific energy dissipation flux (DI_0_/RC) (**d**). Black horizontal bars within the boxplots represent the median. Vertical bars show the maximum and minimum values, and the points outside the box are outlier values. Equal letters above the boxes represent statistically equal means (Tukey’s test, *p* < 0.05).

The inoculation treatments tended to reduce the light absorption flux per active reaction center (ABS/RC) of chlorophyll-*a*, as this index was high only in control plants (2.53) and in plants inoculated with BP33EF (2.58) (Figure 6a). The electron transport flux per reaction center (ET_0_/RC) was also higher in control plants (1.22), but was significantly lower in plants inoculated with BP328EF (1.07) (Figure 6b). The results found for the trapped energy flux per reaction center (TR_0_/RC) were similar to those of ET_0_/RC, with higher means in control plants (1.88) and lower in plants inoculated with BP328EF (1.78) (Figure 6c). The results found for the specific energy dissipation flux at the antenna chlorophyll level (DI_0_/RC) were identical to those found for ABS/RC, denoting higher energy dissipation as heat in control plants (0.55) and plants inoculated with BP33EF (0.54) (Figure 6d).

**Figure 7:**
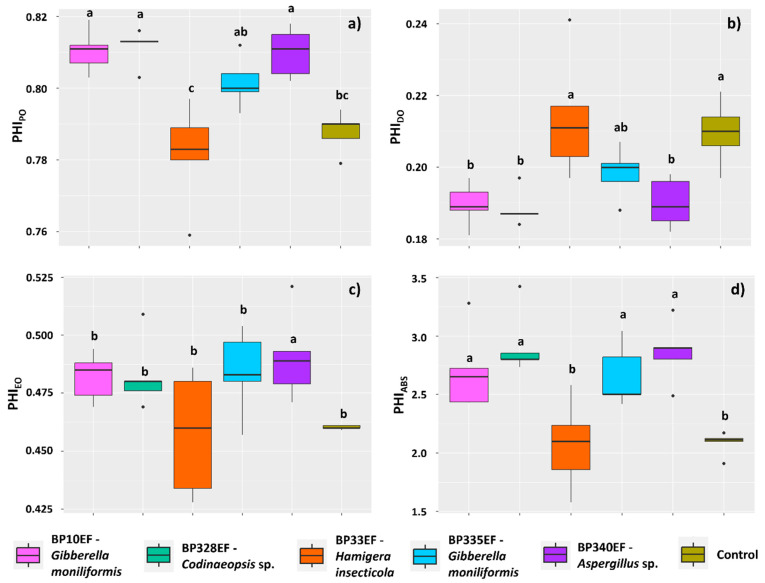
Primary photochemistry by chlorophyll-*a* fluorescence in *Gossypium hirsutum* plants under attack of *Colletotrichum gossypii* var. *cephalosporioides* and inoculated with endophytic fungi. Maximum quantum yield of primary photochemistry: PHI_P0_ (**a**); quantum yield of energy dissipation: PHI_D0_ (**b**); quantum yield of electron transport: PHI_E0_ (**c**); and photosynthetic performance index: PHI_ABS_ (**d**). Black horizontal bars within the boxplots represent the median. Vertical bars show the maximum and minimum values, and the points outside the box are outlier values. Equal letters above the boxes represent statistically equal means (Tukey’s test, *p* < 0.05).

Inoculation with endophytic fungal strains maintained higher levels of maximum quantum yield of primary photochemistry (PHI_P0_) in cotton plants compared with control plants (0.78), except for plants inoculated with BP33EF (0.78) (Figure 7a). The quantum yield of energy dissipation (PHI_D0_), however, showed opposite results, with the highest means in these same treatments, i.e., plants inoculated with BP33EF and control plants (0.21 and 0.20, respectively) (Figure 7b). However, the highest quantum yield of electron transport (PHI_E0_) was found in plants inoculated with BP340EF (0.49) (Figure 7c). The photosynthetic performance index (PI_ABS_) was lower for chlorophylls of plants inoculated with BP33EF (2.07) and control plants (2.08) (Figure 7d).

**Figure 8:**
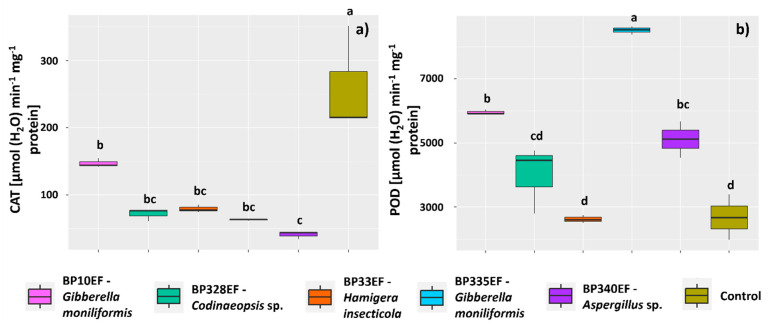
Activity of enzymes of oxidative metabolism in leaves of *Gossypium hirsutum* plants under attack of *Colletotrichum gossypii* var. *cephalosporioides* and inoculated with endophytic fungi. Catalase: CAT (**a**); and peroxidase: POD (**b**). Black horizontal bars within the boxplots represent the median. Vertical bars show the maximum and minimum values, and the points outside the box are outlier values. Equal letters above the boxes represent statistically equal means (Tukey’s test, *p* < 0.05).

Endophytic fungi inoculation in cotton plants exhibiting ramulosis symptoms considerably decreased CAT synthesis in leaf tissues; the highest mean CAT activity was found in control plants (260.27 µmol (H_2_O) min^−1^ mg^−1^ protein) (Figure 8a). The enzyme POD showed opposite results, with lower mean activity in control plants (26,795.18 µmol (H_2_O) min^−1^ mg^−1^ protein) and in plants inoculated with BP33EF (26,212.17 µmol (H_2_O) min^−1^ mg^−1^ protein); this enzyme showed the highest mean activity in leaves of plants inoculated with BP335EF (85,554.79 µmol (H_2_O) min^−1^ mg^−1^ protein) (Figure 8b).

**Figure 9:**
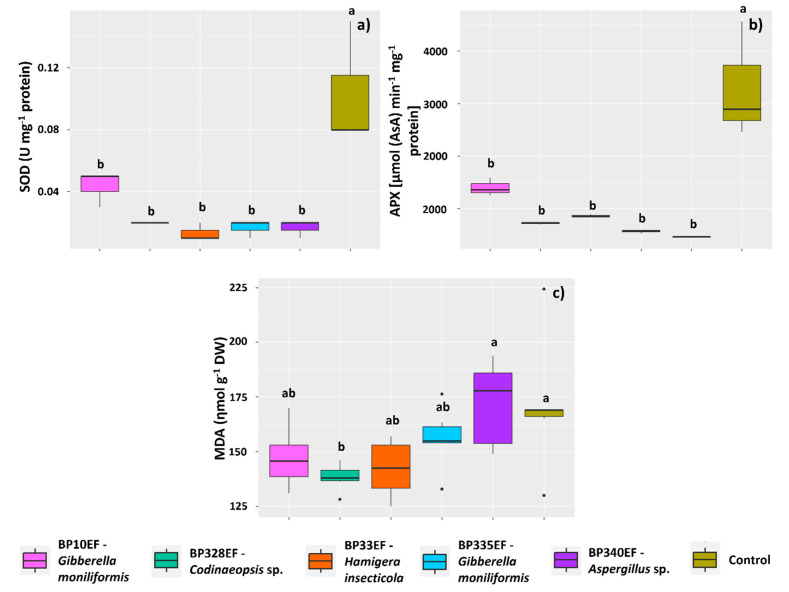
Activity of enzymes of oxidative metabolism and lipid peroxidation in leaves of *Gossypium hirsutum* plants under attack of *Colletotrichum gossypii* var. *cephalosporioides* and inoculated with endophytic fungi. Superoxide dismutase: SOD (**a**); ascorbate peroxidase: APX (**b**); and malondialdehyde: MDA (**c**). Black horizontal bars within the boxplots represent the median. Vertical bars show the maximum and minimum values, and the points outside the box are outlier values. Equal letters above the boxes represent statistically equal means (Tukey’s test, *p* < 0.05).

The activity of the enzymes SOD and APX was similar to that found for CAT; it was stimulated mainly in control plants (0.010 U mg^−1^ protein for SOD and 3309.91 µmol AsA min^−1^ mg^−1^ protein for APX). However, plants in the different inoculation treatments showed no significant differences in the activity of these enzymes (Figure 9a,b). Lipid peroxidation (given by the amount of MDA) in control plants and plants inoculated with BP340EF tended to be higher (179.28 and 165.60 ηmol g^−1^, respectively) (Figure 9c).

**Figure 10:**
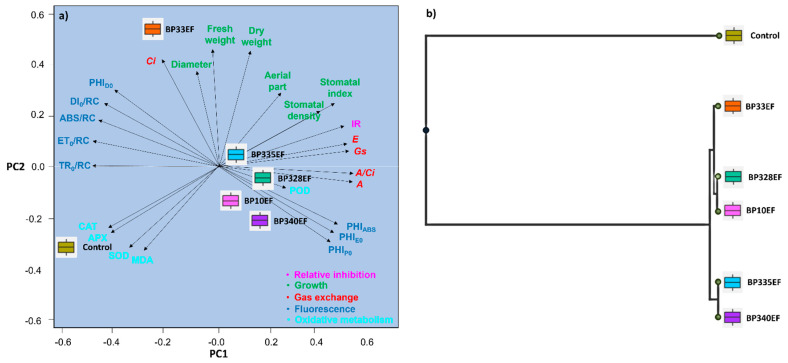
Principal Component Analysis (**a**) and cluster analysis (**b**) for data of plant growth, gas exchanges, fluorescence of chlorophyll-*a*, and oxidative metabolism of *Gossypium hirsutum* plants under attack of *Colletotrichum gossypii* var. *cephalosporioides* and inoculated with endophytic fungal strains (BP10EF: *Gibberella moniliformis*; BP33EF: *Hamigera insecticola*; BP328EF: *Codinaeopsis* sp.; BP335EF: *Gibberella moniliformis*; and BP340EF: *Aspergillus* sp.). In (**a**): relative inhibition index: RII, net photosynthetic rate: *A*; transpiration rate: *E*, intercellular CO_2_ concentration: *Ci*, stomatal conductance: *Gs*, carboxylation efficiency: *A/Ci*, light absorption flux per active reaction center: ABS/RC, electron transport flux per reaction center (ET_0_/RC) at t = 0; trapped energy flux per reaction center (TR_0_/RC) at t = 0; specific energy dissipation flux (DI_0_/RC), maximum quantum yield of primary photochemistry: PHI_P0_, quantum yield of energy dissipation: PHI_D0_, quantum yield of electron transport: PHI_E0_, photosynthetic performance index: PHI_ABS_, catalase: CAT, peroxidase: POD, superoxide dismutase: SOD, ascorbate peroxidase: APX, and malondialdehyde: MDA.

Principal Components 1 and 2 together explained 99.0% of the data variance. This analysis confirmed the trend that oxidative metabolism (activity of enzymes CAT, APX, and SOD) and the cell damage caused by it, given the MDA production, were more active in control plants (Figure 10a). Similarly, control plants and plants inoculated with the endophytic strain BP33EF showed higher results for chlorophyll-*a* fluorescence parameters (ABS/RC, ET_0_/RC, TR_0_/RC, and DI_0_/RC), which are indicators of photochemical stress. Plants inoculated with BP33EF showed the best plant growth performance (stem diameter and shoot fresh and dry weights). Plants inoculated with BP10EF, BP328EF, BP335EF, and BP340EF showed the best photosynthetic indices and photochemical yields. The treatment with BP328EF explained the largest variations in the means of *A*, *E*, *Gs*, and *A*/*Ci*. The cluster analysis showed two stable clusters, confirming the efficiency of fungal inoculation and isolated control plants in the individual cluster (Figure 10b). The similarity between BP328EF and BP10EF grouped these plants in the same cluster, connected to BP33EF. However, divergent means of plant growth and photochemical variables found for BP33EF resulted in a slight separation. Another grouping was established by the similarity between the means found for BP335EF and BP340EF.

## 4. Discussion

### 4.1. Inoculation with Endophytic Fungi Mitigates Physiological and Photochemical Damage by Ramulosis in Cotton Plants

The results corroborate those presented by several studies in the literature, confirming the ability of some endophytic fungi to reduce lesions caused by pathogens through direct antibiosis, production of lytic enzymes, or activation of hormones [1]. However, beneficial microorganisms compete strongly with pathogens for niche colonization and nutrient acquisition [53]. Therefore, endophytic fungal strains can assist in the development of resistance to pests and diseases by affecting the pathogen’s development or reproduction. Studies have confirmed that these fungi can activate ISR (induced systemic resistance) and ASR (acquired systemic resistance) by activating microbial-associated molecular patterns (MAMPs) [54,55,56]. These patterns result in the production of signaling molecules, such as salicylic acid and ethylene. Thus, the colonization of endophytic fungi causes a first activation, making plants more capable of responding to phytopathogenic microorganisms and nematodes [57]. However, the endophytic relationship possibly confers additional defense mechanisms to modulate the plant immune system because of the manipulation of antimicrobial metabolites, either directly, such as alkaloids, or indirectly, such as phytohormones, jasmonic acid, or salicylic acid [55]. 

The present study confirms the potential of endophytic fungi, biotrophic fungi, and/or necrotrophic fungi to mitigate the biotic stress caused by ramulosis in cotton. The tested strains BP10EF and BP335EF of *G. moniliformis* (*Fusarium verticillioides* anamorph) can switch from biotrophic to necrotrophic states, as the biotrophic state can encompass an endophytic condition. Many studies have reported the occurrence of *G. moniliformis* with an endophytic habit [58,59] and confirmed the biotechnological potential of endophytic strains of this species, including for biocontrol [60,61]. The efficacy of BP10EF and BP335EF in controlling ramulosis can be explained by the synthesis of metabolites such as trioleoylglycerol (triolein), naphthoquinone (lawsone), and tricarballylic acid (Fumonisin A–C and P) [60,62]. 

The strains BP340EF (*Aspergillus* sp.) and BP335EF (*G. moniliformis*) showed similar behavior, forming one cluster. Endophytic *Aspergillus* species also produce active molecules associated with the biocontrol functional trait, such as butyrolactones, stigmasterol derivatives, and meroterpenoids e.g., [63,64]. El-hawary et al. [65] reported that different *Aspergillus* species can produce secondary metabolites, including butenolides, alkaloids, terpenoids, cytochalasins, phenalenones, ρ-terphenyls, xanthones, steroids, diphenyl ether, and anthraquinone derivatives, with diverse biological activities, including antifungal and antibacterial effects. Verma et al. [66] indicated that the biosynthesis of silver nanoparticles using an endophytic strain of *Aspergillus clavatus* produces an efficient fungicidal compound for the control of *Candida albicans,* thus reaffirming the importance of species of this genus in biocontrol processes.

The potential of lesser-known fungi was also evaluated. The endophytic strain BP328EF (*Codinaeopsis* sp.) showed a relative inhibition of in vitro growth of *C. gossypii* var. *cephalosporioides* by 81.11%. The genus *Codinaeopsis* encompasses soil fungi capable of synthesizing polyketide codinaeopsin. This metabolite contains an unusual heterocyclic unit that binds indole and decalin fragments and exhibits antimalaria activity [67,68]. Little is known about the effects of inoculating agronomically important plants with fungi of this genus. However, the *Hamigera insecticola* strain (BP33EF) showed potential as a growth promoter in cotton plants, considering its effect on the evaluated biometric characteristics. Studies have confirmed the antifungal potential of species in this genus due to the synthesis of hamigerone and dihydrohamigerone metabolites [69] and silver nanoparticles produced from *Hamigera terricola*, which showed antifungal potential against phytopathogenic species [70].

Besides biocontrol processes and the induction of resistance, studies have confirmed that endophytic fungi can improve plant growth and development [71,72]. Russo et al. [73] showed that species of endophytic entomopathogenic fungi can exhibit traits resulting in the promotion of soybean (*Glycine max*) growth, improving plant biometric development, and increasing grain yield under field conditions. Galeano et al. [74] indicated that the potential of *Aspergillus* species to promote plant growth should be considered. Hamayum et al. [75] showed that *Aspergillus flavus* can mitigate the effects of biotic stress from high temperatures on soybean and sunflower plants; they found significant quantities of indoleacetic acid (IAA), salicylic acid (SA), flavonoids, and phenolic compounds in cultures of this fungus; inoculated plants showed higher dry weight accumulation and chlorophyll contents and lower quantities of abscisic acid (ABA) and proline. This species can mitigate the effects of stress from high salt concentrations and high temperatures on soybean and sunflower plants by regulating endogenous hormones and the antioxidant system [76,77]. Gibberellins produced by *Aspergillus fumigatus* significantly increased shoot length, fresh and dry weights, leaf area, chlorophyll contents, and photosynthetic rate of soybean plants under salt stress [78]. Additionally, Saxena et al. [79] showed that *Aspergillus niger* can promote the growth of *G. max* through phosphate solubilization. In contrast to its saprophytic and pathogenic identity, the ability of filamentous *Aspergillus* fungi to solubilize insoluble phosphates, such as Ca, Fe, and Al phosphates, has stood out [80]. Similarly, Radhakrishnan et al. [81] highlighted the potential of *G. moniliformis* to promote plant growth through phosphate solubilization. They showed that soybean plants inoculated with *G. moniliformis* and subjected to salt stress became more resistant, as this fungus solubilized large quantities of phosphates, reducing oxidative damage and ABA concentrations in leaves, and increasing salicylic acid contents. This explains the growth promotion effects found using the strain BP340EF, mainly in terms of shoot dry weight, stomatal index, carboxylation efficiency, and photochemical yield, as well as the strains BP10EF and BP335EF, mainly in terms of the activation of oxidative stress enzymes.

Fungal inoculation increased stomatal index and density on the adaxial surface of cotton leaves affected by ramulosis, resulting in increased net photosynthetic rate, transpiration, and stomatal conductance with greater carboxylation efficiency. This occurs because stomata serve as an innate immune barrier against infections [82]. Thus, stomatal closure and decreased stomatal index and density are plant strategies to minimize pathogen infection e.g., [83]. Decreases in stomatal density and/or size can significantly affect photosynthesis; thus, plants seem to have a compensation system involving the advantages of reducing gas exchange to prevent the penetration of pathogens [84]. Therefore, as the seeds used in the present study were infected with *C. gossypii* var. *cephalosporioides*, control plants developed leaves with a low stomatal index and density to decrease the possibility of colonization of leaf tissues by *C. gossypii* or other pathogens, which could make the situation of a plant affected by ramulosis even more critical. 

### 4.2. Endophytic Fungi Differentially Affect Growth, Gas Exchange, and Primary Photochemistry of Cotton Plants Affected by Ramulosis 

These results enable discussions about specific functional traits expressed by different lines. Considering the widespread use of biological products to improve disease control and yield in agriculture, species of interest still need to be evaluated and utilized, considering their specific biological functionality, for a better understanding of the effect of mechanisms underlying microbial activity on the plant-microorganism interaction. For instance, the potential of the *H. insecticola* strain BP33EF to promote plant growth was evaluated in the present study. This strain, however, did not alleviate the primary photochemical stress induced by ramulosis in cotton leaves, showing similar ABS/RC and DI_0_/RC to those found in control plants. Consequently, photosynthetic performance (PI_ABS_) was low in these plants. In such cases, compensation studies should be conducted to assess the actual yield gain when using growth-promoting strains that either trigger or do not mitigate metabolic stress processes. 

### 4.3. The Codinaeopsis sp. Strain BP328EF Inhibits the In Vitro Growth of Colletotrichum gossypii var. cephalosporioides, Positively Affects Gas Exchange, and Reduces Chlorophyll-a Photochemical Stress and Lipid Peroxidation

Promising results were found for the use of the *Codinaeopsis* sp. strain BP328EF to mitigate the effects of ramulosis in cotton plants. This genus is not reported in the literature as associated with phytopathogenic characteristics or pathogenicity in animals. *Codinaeopsis* (=*Codinea*) is a polyphyletic genus encompassing phialidic and dematiaceous hyphomycetes [85], known for their intriguing morphology and turbulent taxonomic history. *Codinaea* and its segregates thrive on decomposing plants, rarely occurring as endophytes or plant pathogens. Environmental DNA and ITS sequences indicate their common occurrence in bulk soils. These fungi evolved mainly in Eurasia and the Americas, with subsequent transitions to Africa and Australasia [86]. Little is known about the effects of fungi in this genus on plant growth promotion and stress mitigation. However, the results found in the present study are confirmed by those found by Reis et al. [41], who not only evaluated the potential of *Codinaeopsis* sp. to promote plant growth but also its ability to improve nutrient absorption by *G. max* plants; plant responses regarding chlorophyll index, shoot dry weight, and nutrient concentration (N, P, and Mg) were similar to those of plants treated with a commercial product (Biomaphos^®^, Bioma SA, Quartino, Switzerland) composed of phosphate-solubilizing bacteria. Similarly, an endophytic strain of *Codinaea* sp. significantly affected the elongation of rooted cuttings from different cranberry cultivars [87].

Therefore, field tests should be conducted to assess the effects of applying *Codinaeopsis* sp. to inhibit ramulosis occurrence in cotton plantations. This study contributes new approaches, proposing a biological alternative to improve *G. hirsutum* plants and minimize yield losses due to colonization by *C. gossypii* var. *cephalosporioides*. Thus, mitigating damage caused by endophytic fungi in ramulosis-affected plants encourages the use of new sustainable management practices regarding phytopathogen control in cotton fields. However, the results presented in this study are expected to stimulate prospective studies of endophytic microorganisms in endemic plants of the Cerrado biome, opening prospects for new applications for biological control of pests and diseases in cotton and other important agricultural crops.

## 5. Conclusions

The hypothesis that inoculation of cotton plants with endophytic fungi can attenuate the physiological and photochemical damage caused by ramulosis was confirmed. Overall, endophytic fungi improved plant growth, stomatal index and density, net photosynthetic rate, and carboxylation efficiency while decreasing photochemical and oxidative stresses. Control plants developed leaves with a low adaxial stomatal index and density as a strategy to reduce the likelihood of colonization of leaf tissues by *Colletotrichum gossypii* var. *cephalosporioides* due to the absence of fungal antagonism. The effects of relative inhibition of in vitro growth of *C. gossypii* var. *cephalosporioides* by the activity of the *Codinaeopsis* sp. strain BP328EF were explained as improvements in gas exchange parameters and reductions in chlorophyll-*a* photochemical stress and lipid peroxidation in cotton plants. This study aims to contribute to the development of biological alternatives for improving resistance in cotton (*G. hirsutum*) plants and minimizing yield losses caused by colonization by *C. gossypii*.

## Data Availability

All the data relevant to this manuscript are available on request from the corresponding author.

## References

[1] Tyagi J., Chaudhary P., Jyotsana U.B., Bhandari G., Chaudhary A., Co K.G. (2022). Impact of endophytic fungi in biotic stress management. Plant Protection: From Chemicals to Biologicals.

[2] Kamburova V., Salakhutdinov I., Abdurakhmonov I.Y., Abdurakhmonov I.Y. (2022). Cotton breeding in the view of abiotic and biotic stresses: Challenges and perspectives. Cotton.

[3] Meyer L.A., Dew T. (2021). Cotton and Wool Outlook: December 2021.

[4] Tarazi R., Jimenez J.L.S., Vaslin M.F. (2019). Biotechnological solutions for major cotton (*Gossypium hirsutum*) pathogens and pests. Biotechnol. Res. Innov..

[5] Cox K.L., Babilonia T.W., He P., Shan L. (2019). Return of old foes—Recurrence of bacterial blight and Fusarium wilt of cotton. Curr. Opin. Plant Biol..

[6] Zhu Y., Zhao M., Li T., Wang L., Liao C., Liu D., Li B. (2023). Interactions between *Verticillium dahliae* and cotton: Pathogenic mechanism and cotton resistance mechanism to Verticillium wilt. Front. Plant Sci..

[7] Shete P.P., Kasal Y.G., Perane R.R. (2018). Screening of the cotton genotypes against *Ramularia areola* atk. under field condition. Plant Arch..

[8] Ghaffar A., Mukerji K.G., Garg K.L. (2023). Biological control of sclerotial diseases. Biocontrol of Plant Diseases.

[9] Makwana N., Rawal P. (2022). Cultural, morphological and pathogenic variability of *Colletotrichum gossypii* causing anthracnose of cotton. J. Mycol. Plant Pathol..

[10] Salustiano M.E., Rondon M.N., Abreu L.M., Costa S.S., Costa J.C., Machado L.H. (2014). The etiological agent of cotton ramulosis represents a single phylogenetic lineage within the *Colletotrichum gloeosporioides* species complex. Trop. Plant Pathol..

[11] Moreno-Moran M., Burbano-Figueroa O. (2019). Field resistance of advanced breeding lines of upland cotton to ramulosis caused by *Colletotrichum gossypii* var. *cephalosporioides*. Crop Prot..

[12] Guerra A.M.N.D.M., Rodrigues F.Á., Lima T.C., Berger P.G., Barros A.F., Silva Y.C.R.D. (2014). Photosynthetic capacity of cotton boll rot infected plants and supplied with silicon. Bragantia.

[13] De Araújo A.E., Ferreira A.D.B., Morello C.D.L. Damage caused in cotton by different levels of ramulosis in Brazil. Proceedings of the World Cotton Research Conference-5.

[14] Joshua J., Mmbaga M.T. (2020). Potential biological control agents for soilborne fungal pathogens in Tennessee snap bean farms. HortScience.

[15] Adeleke B.S., Ayilara M.S., Akinola S.A., Babalola O.O. (2022). Biocontrol mechanisms of endophytic fungi. Egypt. J. Biol. Pest Control.

[16] Fontana D.C., De Paula S., Torres A.G., De Souza V.H.M., Pascholati S.F., Schmidt D., Dourado Neto D. (2021). Endophytic fungi: Biological control and induced resistance to phytopathogens and abiotic stresses. Pathogens.

[17] Santra H.K., Banerjee D., Yadav A., Mishra S., Kour D., Yadav N., Kumar A. (2020). Fungal endophytes: A source for biological control agents. Agriculturally Important Fungi for Sustainable Agriculture: Functional Annotation for Crop Protection.

[18] Hassani M.A., Duran P., Hacquard S. (2018). Microbial interactions within the plant holobiont. Microbiome.

[19] Bard N.W., Cronk Q.C., Davies T.J. (2024). Fungal endophytes can modulate plant invasion. Biol. Rev..

[20] Jin L., Yang L., Li W., Xu D., Yang N., Li G., Wan P. (2021). Diversity and biocontrol potential of culturable endophytic fungi in cotton. Front. Microbiol..

[21] Wei F., Zhang Y., Shi Y., Feng H., Zhao L., Feng Z., Zhu H. (2019). Evaluation of the biocontrol potential of endophytic fungus *Fusarium solani* CEF559 against *Verticillium dahliae* in cotton plant. Biomed. Res. Int..

[22] Yuan Y., Feng H., Wang L., Li Z., Shi Y., Zhao L., Zhu H. (2017). Potential of endophytic fungi isolated from cotton roots for biological control against verticillium wilt disease. PLoS ONE.

[23] Gasoni L., de Gurfinkel S. (2009). Biocontrol of *Rhizoctonia solani* by the endophytic fungus *Cladorrhinum foecundissimum* in cotton plants. Australas. Plant Pathol..

[24] Nawaz H.H., Rajaofera M.N., He Q., Anam U., Lin C., Miao W. (2018). Evaluation of antifungal metabolites activity from *Bacillus licheniformis* OE-04 against *Colletotrichum gossypii*. Pestic. Biochem. Physiol..

[25] Yadav L., Yadav N.K., Malik V.K., Yadav P., Yadav N., Vashisth P., Dhariwal S. (2012). Evaluation of biological control agents against *Colletotrichum gossypii* under in vitro condition. Pharma Innov. J..

[26] Ferro H.M., Souza R., Lelis F., Vieira M., Silva J.C.P.D., Medeiros F.H.V.D. (2020). Bacteria for cotton plant protection: Disease control, crop yield, and fiber quality. Rev. Caatinga.

[27] Araújo D.V., Zambenedetti G.B., Dallacort R., Azevedo V.H., Mainardi J.T. (2012). Progresso da ramulose em campo a partir de sementes de algodoeiro inoculadas com *Colletotrichum gossypii* var. cephalosporioides. Trop. Plant Pathol..

[28] Da Silva C.F., Vitorino L.C., Soares M.A., Souchie E.L. (2018). Multifunctional potential of endophytic and rhizospheric microbial isolates associated with *Butia purpurascens* roots. Antonie Van Leeuwenhoek.

[29] Dos Reis J.B.A., do Vale H.M.M., Lorenzi A.S. (2022). Insights into taxonomic diversity and bioprospecting potential of Cerrado endophytic fungi: A review exploring a unique Brazilian biome and methodological limitations. World J. Microbiol. Biotechnol..

[30] Noriler S.A., Savi D.C., Aluizio R., Palacio-Cortes A.M., Possiede Y.M., Glienke C. (2018). Bioprospecting and structure of fungal endophyte communities found in the Brazilian biomes, Pantanal, and Cerrado. Front. Microbiol..

[31] Talhinhas P., Baroncelli R. (2021). Colletotrichum species and complexes: Geographic distribution, host range and conservation status. Fungal Divers..

[32] Chhabra R., Kaur S., Vij L., Gaur K. (2020). Exploring physiological and biochemical factors governing plant pathogen interaction: A review. Int. J. Curr. Microbiol. App. Sci..

[33] Xing J., Li M., Li J., Shen W., Li P., Zhao J., Zhang Y. (2022). Stem canker pathogen *Botryosphaeria dothidea* inhibits poplar leaf photosynthesis in the early stage of inoculation. Front. Plant Sci..

[34] Yang H., Luo P. (2021). Changes in photosynthesis could provide important insight into the interaction between wheat and fungal pathogens. Int. J. Mol. Sci..

[35] Yahya M., Saeed N.A., Nadeem S., Hamed M., Saleem K. (2020). Effect of leaf rust disease on photosynthetic rate, chlorophyll contents, and grain yield of wheat. Arch. Phytopathol. Plant Prot..

[36] Lu Y., Yao J. (2018). Chloroplasts at the crossroad of photosynthesis, pathogen infection and plant defense. Int. J. Mol. Sci..

[37] Gahir S., Bharath P., Raghavendra A.S. (2021). Stomatal closure sets in motion long-term strategies of plant defense against microbial pathogens. Front. Plant Sci..

[38] Grimmer M.K., John Foulkes M., Paveley N.D. (2012). Foliar pathogenesis and plant water relations: A review. J. Exp. Bot..

[39] Goyal A., Parashar M., Nayak R. (2023). Organic cotton and BCI-certified cotton fibres. Sustainable Fibres for Fashion and Textile Manufacturing.

[40] Cabanillas C., Tablada M., Ferreyra L., Pérez A., Sucani G. (2017). Sustainable management strategies focused on native bio-inputs in *Amaranthus cruentus* L. in agroecological farms in transition. J. Clean. Prod..

[41] Reis M.N.O., Vitorino L.C., Lourenço L.L., Bessa L.A. (2022). Microbial inoculation improves growth, nutritional and physiological aspects of *Glycine max* (L.) Merr. Microorganisms.

[42] Strasser R.J., Srivastava A., Tsimilli-Michael M. (2000). The fluorescence transient as a tool to characterize and screen photosynthetic samples. Probing Photosynthesis: Mechanisms, Regulation and Adaptation.

[43] Biemelt S., Keetman U., Albrecht G. (1998). Re-aeration following hypoxia or anoxia leads to activation of the antioxidative defense system in roots of wheat seedlings. Plant Physiol..

[44] Havir E.A., McHale N.A. (1987). Biochemical and developmental characterization of multiple forms of catalase in tobacco leaves. Plant Physiol..

[45] Nakano Y., Asada K. (1981). Hydrogen peroxide is scavenged by ascorbate-specific peroxidase in spinach chloroplasts. Plant Cell Physiol..

[46] Fang W.-C., Kao C.H. (2000). Enhanced peroxidase activity in rice leaves in response to excess iron, copper and zinc. Plant Sci..

[47] Bradford M.M. (1976). A rapid and sensitive method for the quantitation of microgram quantities of protein utilizing the principle of protein-dye binding. Anal. Biochem..

[48] Giannopolitis C.N., Ries S.K. (1977). Superoxide dismutases: I. Occurrence in higher plants. Plant Physiol..

[49] Buege J.A., Aust S.D., Qin P.Z. (1978). Microsomal lipid peroxidation. Methods in Enzymology.

[50] R Core Team (2024). R: A Language and Environment for Statistical Computing [Software].

[51] Sokal R.R., Rohlf F.J. (1962). The comparison of dendrograms by objective methods. Taxon.

[52] Garcia-Vallve S., Palau J., Romeu A. (1999). Horizontal gene transfer in glycosyl hydrolases inferred from codon usage in *Escherichia coli* and *Bacillus subtilis*. Mol. Biol. Evol..

[53] Haas D., Keel C. (2003). Regulation of antibiotic production in root-colonizing *Pseudomonas* spp. and relevance for biological control of plant disease. Annu. Rev. Phytopathol..

[54] Lu H., Wei T., Lou H., Shu X., Chen Q. (2021). A critical review on communication mechanism within plant-endophytic fungi interactions to cope with biotic and abiotic stresses. J. Fungi.

[55] Yan L., Zhu J., Zhao X., Shi J., Jiang C., Shao D. (2019). Beneficial effects of endophytic fungi colonization on plants. Appl. Microbiol. Biotechnol..

[56] Latz M.A., Jensen B., Collinge D.B., Jørgensen H.J. (2018). Endophytic fungi as biocontrol agents: Elucidating mechanisms in disease suppression. Plant Ecol. Divers..

[57] Poveda J. (2021). Trichoderma as biocontrol agent against pests: New uses for a mycoparasite. Biol. Control.

[58] Eo J.K., Choi M.S., Eom A.H. (2014). Diversity of endophytic fungi isolated from Korean ginseng leaves. Mycobiology.

[59] Desjardins A.E., Busman M., Muhitch M., Proctor R.H. (2007). Complementary host–pathogen genetic analyses of the role of fumonisins in the *Zea mays–Gibberella moniliformis* interaction. Physiol. Mol. Plant Pathol..

[60] Kim J.W., Ryu J., Shim S.H. (2018). Chemical investigation on an endophytic fungus *Gibberella moniliformis* JS1055 derived from a halophyte *Vitex rotundifolia*. Nat. Prod. Sci..

[61] Sarang H., Rajani P., Vasanthakumari M.M., Kumara P.M., Siva R., Ravikanth G., Uma Shaanker R. (2017). An endophytic fungus, *Gibberella moniliformis* from *Lawsonia inermis* L. produces lawsone, an orange-red pigment. Antonie Van Leeuwenhoek.

[62] Proctor R.H., Brown D.W., Plattner R.D., Desjardins A.E. (2003). Co-expression of 15 contiguous genes delineates a fumonisin biosynthetic gene cluster in *Gibberella moniliformis*. Fungal Genet. Biol..

[63] Ibrahim S.R., Elkhayat E.S., Mohamed G.A., Khedr A.I., Fouad M.A., Kotb M.H., Ross S.A. (2015). Aspernolides F and G, new butyrolactones from the endophytic fungus *Aspergillus terreus*. Phytochem. Lett..

[64] Bai Z.-Q., Lin X., Wang J., Wang Y., Zhou X., Yang B., Liu J., Wang Y., Liu Y. (2014). New phenyl derivatives from endophytic fungus *Aspergillus flavipes* AIL8 derived of mangrove plant *Acanthus ilicifolius*. Fitoterapia.

[65] El-Hawary S.S., Moawad A.S., Bahr H.S., Abdelmohsen U.R., Mohammed R. (2020). Natural product diversity from the endophytic fungi of the genus Aspergillus. RSC Adv..

[66] Verma V.C., Kharwar R.N., Gange A.C. (2010). Biosynthesis of antimicrobial silver nanoparticles by the endophytic fungus *Aspergillus clavatus*. Nanomedicine.

[67] Ramanathan M., Tan C.J., Chang W.J., Tsai H.H.G., Hou D.R. (2013). Synthesis of the decalin core of codinaeopsin via an intramolecular Diels–Alder reaction. Org. Biomol. Chem..

[68] Kontinik R., Clardy J. (2008). Codinaeopsin, an antimalarial fungal polyketide. Org. Lett..

[69] Breinholt J., Kjoer A., Olsen C.E., Rassing B.R. (1997). Hamigerone and dihydrohamigerone: Tvvo acetate-derived, antifungal metabolites from Hamigera. Acta Chem. Scand..

[70] Mistry H., Thakor R., Polara H., Shah T., Bariya H., Kaneria M., Rakholiya K., Egbuna C. (2024). Biogenically efficient production and characterization of silver nanoparticles using the marine fungus *Hamigera terricola* along with their antimicrobial and antioxidative efficacy. Nanotechnology and In Silico Tools.

[71] Baron N.C., Rigobelo E.C. (2022). Endophytic fungi: A tool for plant growth promotion and sustainable agriculture. Mycology.

[72] Poveda J., Eugui D., Abril-Urías P., Velasco P. (2021). Endophytic fungi as direct plant growth promoters for sustainable agricultural production. Symbiosis.

[73] Russo M.L., Pelizza S.A., Vianna M.F., Allegrucci N., Cabello M.N., Toledo A.V., Scorsetti A.C. (2019). Effect of endophytic entomopathogenic fungi on soybean *Glycine max* (L.) Merr. growth and yield. J. King Saud Univ.-Sci..

[74] Galeano R.M.S., Franco D.G., Chaves P.O., Giannesi G.C., Masui D.C., Ruller R., Zanoelo F.F. (2021). Plant growth-promoting potential of endophytic *Aspergillus niger* 9-p isolated from native forage grass in Pantanal of Nhecolândia region, Brazil. Rhizosphere.

[75] Hamayun M., Hussain A., Khan S.A., Iqbal A., Lee I.J. (2019). *Aspergillus flavus* promoted the growth of soybean and sunflower seedlings at elevated temperature. BioMed Res. Int..

[76] Ismail, Hamayun M., Hussain A., Iqbal A., Khan S.A., Lee I.J. (2020). *Aspergillus niger* boosted heat stress tolerance in sunflower and soybean via regulating their metabolic and antioxidant system. J. Plant Interact..

[77] Asaf S., Hamayun M., Khan A.L., Waqas M., Khan M.A., Jan R., Hussain A. (2018). Salt tolerance *of Glycine max* L. induced by endophytic fungus *Aspergillus flavus* CSH1, via regulating its endogenous hormones and antioxidative system. Plant Physiol. Biochem..

[78] Khan A.L., Hamayun M., Kim Y.H., Kang S.M., Lee J.H., Lee I.J. (2011). Gibberellins producing endophytic *Aspergillus fumigatus* sp. LH02 influenced endogenous phytohormonal levels, isoflavonoids production and plant growth in salinity stress. Process Biochem..

[79] Saxena J., Rawat J., Sanwal P. (2016). Enhancement of growth and yield of *Glycine max* plants with inoculation of phosphate solubilizing fungus *Aspergillus niger* K7 and biochar amendment in soil. Commun. Soil Sci. Plant Anal..

[80] Nayak S., Samanta S., Mukherjee A.K. (2020). Beneficial role of *Aspergillus* sp. in agricultural soil and environment. Frontiers in Soil and Environmental Microbiology.

[81] Radhakrishnan R., Khan A.L., Kang S.M., Lee I.J. (2015). A comparative study of phosphate solubilization and the host plant growth promotion ability of *Fusarium verticillioides* RK01 and *Humicola* sp. KNU01 under salt stress. Ann. Microbiol..

[82] Melotto M., Zhang L., Oblessuc P.R., He S.Y. (2017). Stomatal defense a decade later. Plant Physiol..

[83] Murray R.R., Emblow M.S., Hetherington A.M., Foster G.D. (2016). Plant virus infections control stomatal development. Sci. Rep..

[84] Muir C.D. (2020). A stomatal model of anatomical tradeoffs between gas exchange and pathogen colonization. Front. Plant Sci..

[85] Maire R. (1937). Fungi Catalaunici: Series Altera. Contributions à L’étude de la Flore Mycologique de la Catalogne.

[86] Réblová M., Kolařík M., Nekvindová J., Réblová K., Sklenář F., Miller A.N., Hernández-Restrepo M. (2021). Phylogenetic reassessment, taxonomy, and biogeography of *Codinaea* and similar fungi. J. Fungi.

[87] Salhi L.N., Bustamante Villalobos P., Forget L., Burger G., Lang B.F. (2022). Endosymbionts in cranberry: Diversity, effect on plant growth, and pathogen biocontrol. Plants People Planet.

